# John Walker's Reply to James Moore, on His Mis-Statements Respecting the Vaccine Establishments in the Metropolis, and Their Officers or Servants, Both Living and Dead

**Published:** 1818-06

**Authors:** 


*John Walker's Reply to James Moore, on his Mis-statements respecting the Vaccine Establishments in the Metropolis, and their Officers or Servants, both Living and Dead. Octavo. pp.* 110. *London*, 1818.

We felt it our duty, in our late review of Mr. Moore's work, to praise the poignancy of his playful satire, the shrewdness of his expression, and the caricatural facility of the touches with which he hits off men and manners,—touches that, for fidelity and strength, have scarcely been surpassed by those of Theophrastus or La Bruyere. But though much taken with his wit, we are not prepared to say he has not carried it too far. Indeed, we think, his sarcastic humour may be likened unto that great (though rare) blessing—a good razor: as the latter deftly doeth its tonsorial office, and plungeth with ready and remorseless edge through the bodies of opposing pimples of all sorts and conditions, whether great or small, meek or fiery;—so the former, handled by this historian of vaccination, spares nothing that comes in its way, from the wrong-headed anti-vaccinist, or fee-hunting physician, to the "unctuous citizen"; from the stagnant atmosphere and sober society of Gosport or Bristol, to the airy downs and ultra-fashionable folks of Cheltenham or Bath; from the "hot Hibernian, not yet cooled down to the medical point," to the peace-professing member of the "Society of Friends."

We are somewhat in love with the above simile, because it is a *bright* one; and it farther holds in this, that whereas some of the aforesaid pimples often raise their heads again, as it were to protest against

"The deep damnation of their *taking off*,"

and occasion no little pain and smart in the sequel;—so also the victims of the satirical razor are sometimes apt to vociferate under the keen edge of its operation as loudly as the poor Frenchman "qu'on rasoit pour l'amour de Dieu," and afterwards give vent to their agony and their anger by abusing the hand that weilded the painful instrument, whose very sharpness caused it to work them the greater woe.

Without further metaphor, the simple matter is, that Mr. Moore has thrown out his gibes and jokes with rather too regardless a hand, and has experienced the serious truth, that joking, like *lending*,

"Oft loses both itself and friends."

He has displeased many and incensed some: amongst the latter it too plainly appears our author is to be found. Mr. M. has irritated him in the inward man, and moved the quiescent spirit of the worthy Quaker to a most resentful reply.

Let us now analyze that reply.

When Owen Glendower boasted that he could "call spirits from the vasty deep," somebody asked, "Would they come? Dr. Walker has made a similar sort of call, and we would beg, respectfully, to put to him a similar kind of question. In the following passage, he conjures peers, potentates, townships, churchmen, sectarians, and *every other* denomination of persons, in all countries and climes, to come to his aid, and make common cause with him against his antagonist; yet we are much deceived indeed, if they will listen to the distant summons, or rise to his assistance in the way he wishes.

"People of Portsmouth, Portsea, Gosport, &c.! Inhabitants of Bath, Bristol, London, of India and the West Indies! Dignitaries, Dowagers, Sovereigns of other countries! The whole female sex, [*mercy on us!*] and the male too! People of the world at large! Poets, pastoral or dramatic; Fanatics, Episcopalians, Quakers, Methodist Preachers, Free-thinkers! But, most of all, ye sailors of the British fleets, go purchase the book, and contemplate your pictures! The mirror of Moore will most completely reveal you to yourselves.—e.g."

'As Lord Keith, the admiral, had heard from home the good effects of the vaccine, he permitted a trial to be made. It may be easily conceived that this proposal was at first little relished by seamen, a class of men peculiaily inconsiderate of futurity. But as Walker, one of the travelling preachers, had a strange appearance, uncouth manners, and unintelligible arguments, he made many converts in the fleet.' p. 8.

We extract the above passage, not only as being a fair specimen of Dr. Walker's style, in which there is a singular mixture of inflation and drollery, but as containing part of the provocation, on Mr. Moore's side, that has given cause to the present Reply. The remaining part of that provocation we shall also extract, for the reader's amusement.

'Though the question was completely foreign to polemics, yet the religious professions of the subscribers seemed to influence their votes; for a great majority of the Episcopalians, Dissetners, and Free-thinkers, agreed in the deposition of Dr. Walker; but the Quakers were the staunch supporters of a delinquent of their own sect. In this beneficent society they were even at first somewhat numerous; and many others of this persuasion had latterly hastened to subscribe, whose charity was simultaneous with the perilous condition of Walker. At all general meetings their broad brimmed hats shaded the boards; for their schismatic assiduity was most conspicuous, though their primitive meekness was indiscernable. In support of their friend, they argued slily, and voted almost unanimously. Yet, in spite of this contentious pertinacity, the turbulent quaker, on the motion of Dr. Jenner, was dismissed from his office, and peace was restored." *Mr. Moore's History, p.* 207.

"James Moore knows that this statement is not true, [*Here follows an immense note for which we have no room*] for he himself seconded the motion for my dismissal at a general court, July 25, 1806, and the decision was exactly the reverse of what he asserts." p. 46.

We know nothing, and care just as little, about the transaction referred to, and therefore pretend not to say, whether Mr. Moore's or Dr. Walker's be the more correct of the two. But granting that the latter were right, and admitting that the provocation on the part of the former is considerable, we cannot see how the calumnious scurrility that pervades this Reply can any way be justified. From p. 61 to 64 he makes a gross attack on Mr. Moore's personal and political character; the former is traduced on the authority of some gossiping lady of quality, and the latter on Dr. W's own: it must be admitted that the authority, in both cases, is nigh upon a par.

Surely Mr. M. will not be ill-advised enough to take any notice of this production: certainly he needs not, for it carries with it its own destruction, *if let alone.* The polluted waters of private scandal, when suffered to stagnate, soon become disgusting to every one, simply from the intestine re-action of their own impure and malignant ingredients.

We have another charge to make against Dr. Walker; viz. that he often betrays the most odious nationality, (which, we think, is tantamount to saying that he betrays an inconceivably little mind); and talks, forsooth, as if virtue depended on local demarcations, or, as if scoundrels, as well as talents, were not indigenous to both sides of the Tweed. Verily for *this,* also, he shall have his reward, in the contempt of the enlightened part of his readers.

But animosity and nationality are not all that this singular book contains: it is stored with sundry other matters. In fact it is a real *Olla Podrida,* and bears a strong resemblance to those savoury dishes which some skilful house-wives (more eminent for their *saving* knowledge than for their cleanliness) are said to make up from scraps of beef, scrags of mutton, scrapings of cupboards, and other little odds and ends. For instance, he tells us, that like Ulysses, he has been a great traveller in his youth; that he admired the French revolution, and sustained the fraternal hug of worthy citizen Gregoire and Co.; that one *Count Beccaria* wrote him a letter full of sentimentality and illumination; and that some *Lord* [the Lord knows who !] complimented the grandeur and originality of his views! Moreover, he sets forth, how, while in Paris, he was turned out of the hall of the Council of Ancients for refusing to take off his hat, but that he afterwards gave them a wipe for their impolite behaviour, in a long letter, wherein, by way of episode (for the reader must know Dr. W. is remarkably fond of episodes,) he introduced the *projet* of a law for preventing the maltreatment, and ameliorating the condition of baited bulls, horses, dogs, and dancing bears! All these, and sundry other curious particulars, too tedious to mention in this analytical sketch, are detailed at full length; and he that listeth to read them may do so, in the work itself, for the small sum of three shillings and sixpence.

We shall hardly be suspected of partiality when we say that both Dr. Walker and Mr. Moore are perfectly unknown to us, except through their writings; and that we are quite unconcerned in this controversy. The former is, very likely, a worthy man, but, as a writer, we sincerely think his judgment not good, and his taste execrable. The only tolerable portion of his book (the biography of Dr. Woodville,) we have already presented to our readers in a former number; and even that portion is not a little disfigured by the pomposity and peculiarity of the author's manner.

